# Dietary changes in nutritional studies shape the structural and functional composition of the pigs’ fecal microbiome—from days to weeks

**DOI:** 10.1186/s40168-017-0362-7

**Published:** 2017-10-27

**Authors:** Bruno Tilocca, Katharina Burbach, Charlotte M. E. Heyer, Ludwig E. Hoelzle, Rainer Mosenthin, Volker Stefanski, Amélia Camarinha-Silva, Jana Seifert

**Affiliations:** 1 Institute of Animal Science, University of Hohenheim, Emil-Wolff-Str. 6-10, 70593 Stuttgart, Germany; 2 Department of Agricultural, Food and Nutritional Science, University of Alberta, Edmonton, Canada

## Abstract

**Background:**

The possible impact of changes in diet composition on the intestinal microbiome is mostly studied after some days of adaptation to the diet of interest. The question arises if a few days are enough to reflect the microbial response to the diet by changing the community composition and function. The present study investigated the fecal microbiome of pigs during a time span of 4 weeks after a dietary change to obtain insights regarding the time required for adaptation. Four different diets were used differing in either protein source (field peas meal vs. soybean meal) or the concentration of calcium and phosphorus (CaP).

**Results:**

Twelve pigs were sampled at seven time points within 4 weeks after the dietary change. Fecal samples were used to sequence the 16S rRNA gene amplicons to analyse microbial proteins via LC-MS/MS and to determine the SCFA production. The analysis of OTU abundances and quantification values of proteins showed a significant separation of three periods of time (*p* = 0.001). Samples from the first day are used to define the ‘zero period’; samples of weeks 1 and 2 are combined as ‘metabolic period’ and an ‘equilibrium period was defined based on samples from weeks 3 and 4. Only in this last period, a separation according to the supplementation of CaP was significantly detectable (*p* = 0.001). No changes were found based on the corn-soybean meal or corn-field peas administration. The analysis of possible factors causing this significant separation showed only an overall change of bacterial members and functional properties. The metaproteomic approach yielded a total of about 9700 proteins, which were used to deduce possible metabolic functions of the bacterial community.

**Conclusions:**

A gradual taxonomic and functional rearrangement of the bacterial community has been depicted after a change of diet composition. The adaptation lasts several weeks despite the usually assumed time span of several days. The obtained knowledge is of a great importance for the design of future nutritional studies. Moreover, considering the high similarities between the porcine and human gastrointestinal tract anatomy and physiology, the findings of the current study might imply in the design of human-related nutritional studies.

**Electronic supplementary material:**

The online version of this article (10.1186/s40168-017-0362-7) contains supplementary material, which is available to authorized users.

## Background

The intestinal microbiota is involved in a variety of physiological processes of primary importance for the host metabolism and growth, such as nutrient absorption, metabolism and utilisation [1, 2]. Other vital processes including host immune-modulation and prevention from metabolic and neoplastic diseases have often been related to the intestinal microbiota composition and activity [3].

Due to these important implications, several investigations of the microbiota are nowadays carried out on humans and other animal models, in the attempt to elucidate the onset mechanisms of impactful pathological conditions such as obesity, inflammatory bowel disease, diarrhea, necrotizing enterocolitis and many others [3, 4]. In the recent years, animal scientists started to perform in-depth microbiota investigations for the optimisation of the animal husbandry strategies as well as the improvement of animal’s health status [2, 5].

Diet represents one of the major environmental factors shaping the intestinal microbiota. Here, a varying ratio of carbohydrates and proteins or a change of the source of these basal feed components were important key factors [6]. Besides these main feed components, minerals and trace elements are known to influence the intestinal microbiota [7]. Due to the intrinsic incapability for an autonomous phosphorus (P) uptake, standard pig diet contains a supplemental level of calcium and phosphorus (CaP) [8]. This results in a higher excretion of the respective minerals, contributing to the environmental problem of water eutrophication, and besides, being responsible for a useless raise of husbandry costs and a waste of valuable P resources [9, 10]. These reasons pose the need to reduce the P excretion by reducing the dietary CaP-supplementation. However, changes in the diet formulation may be associated with the alteration of microbiota composition and activity due to the needs to fulfil nutritional requirements or by the alteration of the physicochemical condition of the gut lumen resulting in an awkward milieu for microbial colonisation and growth [9, 11, 12]. Studies performed in rats which were kept on a diet with high CaP levels indicated an increased amount of beneficial lactobacilli and an increased resistance to intestinal pathogen colonisation [13]. These results are in agreement with other studies performed at the luminal [9, 11] and mucosal [14] level of the pig’s gastrointestinal tract.

Although various studies investigated the dietary-induced modulation of the pigs intestinal microbiota composition and activity, it is still being discussed how and how long the microbiota adaptation process lasts. This fact is of great importance for nutritional studies including the translational research for human health. Pigs and rodents are the foremost microbiota models for translational studies into the human field [15]. Pigs resemble humans more than rodents in terms of dietary regimen, gastrointestinal tract anatomy, physiology and nutrient digestibility [16, 17]. Moreover, similar to the human intestinal microbiota, the intestinal microbiota of pigs is primarily composed of the phyla of Firmicutes and Bacteroidetes with a varying composition depending on the concerned section along the gastrointestinal tract [2].

So far, to the best of our knowledge, no studies were performed to investigate the progressive adaptation of the pigs’ intestinal microbiota challenged by feeding different experimental diets. Therefore, it was our objective to investigate the expected gradual adaptation of the fecal microbiota over an experimental period of 4 weeks. 16S rRNA gene sequencing along with a metaproteomic approach were employed to provide an exhaustive description of the structural and functional changes of the intestinal microbiota triggered by the experimental treatments. The experimental diets fed varied in the composition of the protein source and the amount of supplemented CaP. The results of this study provide novel insights into the structural and functional changes during the adaptation periods and show that weeks rather than days are required to observe a significant change in the microbial community composition and function.

## Methods

### Animal experiment and experimental diets

Twelve pigs (German Landrace x Piétrain, initial body weight 54.7 kg ± 4.1 kg) were randomly assigned to four experimental diets. The diets were formulated to meet or exceed the animal’s nutrient requirements and differed among each other in the protein source and the CaP levels. Two out of four diets contained low digestible (LD) corn-field pea meal as a protein source whereas the remaining two diets comprised highly digestible (HD) corn-soybean meal as a protein source. Each of these dietary groups was further supplied with high and low CaP levels. Diets with high and low CaP levels were formulated to contain 120 and 66% of the requirements for 50–75 kg pigs (NRC, 2012). In all diets, the Ca:P ratio was kept at 2:1 constantly. Gross energy content of the corn-field peas-based diets was 18.83 MJ/kg whereas the energetic content of the corn-soybean based diets was 19.60 MJ/kg. Further details on the animal experiments and experimental diets are provided in [18].

All animals were initially fed with a conventional diet until week 12 of age. At week 13 of age, the animals were randomly assigned to the four experimental diets (three animals per diet) which were fed until week 20 of age. The pigs were individually allocated in pens, and fecal samples were collected constantly, before and during the experimental treatments. Eighty-four fecal samples across the whole experimental time span were selected for the investigation of the fecal microbiota adaptation to the experimental diets. Samples were collected on ice and immediately stored at − 80 °C until subsequent analysis. Details on the experimental trial and sampling time are reported in Table 1.

**Table 1:** Fecal samples were collected seven times across an experimental time span of 4 weeks and independently subjected to both 16S rRNA gene sequencing and metaproteomic investigation (X)

Animal number	Diet		Experimental period						
		week	0	1			2	3	4
		day	3	8	10	12	17	25	32
01	R4		X	X	X	X	X	X	X
03	R3		X	X	X	X*	X	X	X
04	R1		X	X	X	X	X	X	X
05	R4		X	X	X	X	X	X	X
06	R2		X	X	X	X	X	X	X
07	R1		X	X	X	X	X*	X	X
08	R4		X	X	X	X	X	X	X
11	R2		X	X	X	X	X	X	X
12	R3		X*	X	X	X	X	X	X
13	R1		X	X	X	X	X	X	X
15	R2		X	X	X	X	X	X	X
16	R3		X	X	X	X	X	X	X

Failed DNA analyses are indicated by X*. *R1* corn-soybean, high-digestible (HD), high CaP; *R2* corn-soybean, HD, low CaP; *R3* corn-field pea, low digestible (LD), high CaP; *R4* corn-field pea, LD, low CaP

### DNA extraction and amplicon sequencing

In accordance with a previous study [19], the FastDNA™ SPIN Kit for Soil (MP Biomedicals, Heidelberg, Germany) was used for DNA extraction by following the manufacturer’s instruction with slight modifications. Briefly, 250 mg of feces were added to a Lysing Matrix E tube supplied with the provided buffers. Bead beating was performed twice in a Fast Prep®-24 Instrument (6 m/s, 40 s). Cell lysates were separated by centrifugation (14,000×*g*, 15 min) and proteins were precipitated from the supernatant. DNA was bound to a silica matrix on a spin filter and eluted with 55 °C pre-warmed DES water. DNA extracts were quantified in a NanoDrop instrument (Thermo Fisher Scientific, Waltham, MA).

The V1-V2 region of the 16S rRNA gene was amplified once for each sample and sequenced as previously described [20]. The primer pair 27F-338R was used to amplify the target region, with a slightly modified sequence of the primer 27F (AGRGTTHGATYMTGGCTCAG). Obtained amplicons were verified by agarose gel electrophoresis, purified and normalised with SequalPrep™ Normalization Plate kit (Invitrogen, Carlsbad, CA, USA). Libraries were pooled by index, purified with MinElute PCR Purification Kit (Qiagen, Hilden, Germany) and quantified by using QuantiFluor® dsDNA System (Promega, Madison, USA). Amplicons were sequenced on an Illumina MiSeq in paired-end mode (2 × 250 base pairs). Sequence reads were quality filtered and assembled using Mothur software package [21]. Sequences were quality filtered by excluding reads that had an average quality score lower than 20, a total length of more than 355 base pairs (bp), any primer or barcode mismatch, more than eight homopolymer stretches or an N character. Reads were checked for chimeras and were clustered into operational taxonomical units (OTUs) at 97% identity [22]. OTUs appearing only once across the samples as well as those with less than 10 reads each were manually deleted. The remaining OTUs were finally assigned to the closest taxonomical representative using seqmatch from RDP [22].

OTU abundances were subjected to statistical investigation using Primer6 v.6 statistical software (PRIMER-E, Plymouth, UK) [23]. Prior to statistical analysis, the amplicon sequencing data was standardised by abundances of all sequences, square root transformed and the principal coordinate analysis (PCoA) was calculated on the basis of the Bray-Curtis dissimilarity matrix. A Good’s coverage index greater than 98% indicated sufficient sampling of our data and adequate depth. Statistical differences across time points over the experimental time frame and between diets were calculated by performing ANOVA with permutations (PERMANOVA).

### Sample preparation for LC-MS/MS analyses

Procedures for sample preparation, including protein extraction and the in-gel digestion of the proteins were performed as previously described in Tilocca et al. [12].

Tryptic peptides were purified and desalted by using self-assembled C18 Stage Tips [24]. Tips containing the C18 membranes with the bounded peptide mixture were stored at − 20 °C and resuspended in 5% acetonitrile (5% ACN/ 0.1% TFA) prior to the LC-MS/MS measurements.

### LC-MS/MS analysis

A volume of 1.5 μL of the resuspended peptides mixture was measured by using a Q-Exactive HF mass spectrometer (Thermo Fisher Scientific, Darmstadt, Germany) faced with an EasyLC 1000 nano-UHPLC (Thermo Fisher Scientific, Darmstadt, Germany) as described previously [25]. Separation of peptides was performed on a 20-cm fused silica column of 75-μm inner diameter (Proxeon Biosystems). The column has been in-house packed with reversed-phase ReproSil-Pur 120 C18-AQ 1.9 μm resin (Dr. Maisch GmbH, Ammerbuch, Germany). Peptides were loaded onto the column in solvent A (0.1% formic acid) at a flow rate of 500 nl/min and subsequently eluted with an 87-min segmented gradient of 10–50% HPLC solvent B (80% ACN in 0.1% formic acid).

The MS/MS instrument was set to positive ion mode. Full scans were acquired in the mass range from m/z 300 to 1650 in the Orbitrap mass analyser at a resolution of 120,000 followed by HCD fragmentation of the 12 most intense precursor ions. High-resolution MS/MS spectra were acquired with a resolution of 30,000. The target values were 3 * 10^6^ charges for the MS scans and 1 * 10^5^ charges for the MS/MS scans with a maximum fill time of 25 and 45 ms, respectively. Fragmented masses were excluded for 30 s after MS/MS. Spectra de-noising was performed prior to peptides identification by considering only the top 12 peaks in a window of 100 Da width.

### Bioinformatics analysis of protein data

Out of the total LC-MS/MS raw data inventory, a restricted number of samples were selected for a preliminary investigation of the bacterial protein composition. Selected samples were representative of the potential variability induced by the experimental treatments and the potential variability across the experimental time frame.

Sorted raw data were processed through Thermo Proteome Discoverer software (v.1.4.1.14) and searched against NCBInr bacteria database (release 19 October 2015) in order to evaluate the overall taxonomic composition and to export a consensus fasta database. Methionine oxidation was set as variable modification and carbamidomethylation of cysteine as fixed modification. The Mascot significance threshold was set to 0.05, and a filter considering only entries with at least one peptide per protein was chosen. All other filters and settings of the software were kept as default, including protein grouping with peptide confidence set on “high” and delta Cn of 0.1. The Percolator node supporting a strict maximum parsimony principle was activated with a false discovery rate of 1%.

The consensus protein fasta database obtained from the previous Proteome Discoverer processing of the raw files was employed as an *in-house* database (14,535 entries) for a second search performed on the MaxQuant software. The use of a custom database for processing the whole raw data inventory maximises the protein identification rate and reduces the false discovery rate by including only protein entries that exclusively belong to the bacterial specimen of our interest [26]. Additionally, an independent database-dependent search of all raw files was performed against UniProtKB database (release March 2016) *Sus scrofa* (UniProt ID 9823; 61,019 entries). MaxQuant software (v.1.5.3.8), set on LFQ modality, was used for peptide identification and protein inference. Cysteine carbamidomethylation was set as fixed modification and methionine oxidation as variable modification. Two missed cleavage sites were allowed for in silico protease digestion and peptides had to be fully tryptic. All other parameters of the software were set as default, including a peptide and protein FDR < 1%, at least 1 peptide per protein, precursor mass tolerance of 4.5 ppm after mass recalibration and a fragment ion mass tolerance of 20 ppm.

Taxonomic information was inferred according to the protein description obtained from the MaxQuant search results. These in turn were gathered from the protein annotation of the chosen database (i.e. NCBInr). Identified proteins were functionally classified into COG and KEGG categories via WebMGA [27] with an e-value cut-off of 10^−3^ considering exclusively the best hits. Qualitative evaluation of the resulting DNA-based and metaproteomic datasets have been performed by sorting the OTUs and protein accession numbers into the respective adaptation period. Comparisons between adaptation periods were performed for each dataset and presented as Venn diagrams using the Venny online tool. Protein abundance indexes of the identified proteins (LFQ values) were subjected to statistical investigation through the use of Primer6 v.6 statistical software (PRIMER-E, Plymouth, UK) [23]. Principal coordinate analysis (PCoA) was calculated on the basis of the Bray-Curtis dissimilarity matrix which in turn was calculated on the square root transform of the protein LFQs [28]. Statistical differences across time points over the experimental time frame and between diets were calculated by performing a PERMANOVA. Similarity percentage analysis (SIMPER) was also performed in order to isolate proteins driving dissimilarities between adaptation periods [29]. Heat maps visualising microbial community composition across the adaptation periods and functional classification of the identified proteins were drawn using heatmap.2 provided by the gplots package [30] implemented in R v.3.1.2 software (http://www.R-project.org).

### Analysis of microbial metabolites

Short-chain fatty acids (SCFAs) were analysed by direct measurements of feces. Samples were prepared as previously described [31] followed by gas chromatography (GC) with flame ionization detector (HP 6890 Plus; Agilent, Waldbronn, Germany) measurements using fatty acids (GC grade; Fluka, Taufkirchen, Germany) as internal standards [32]. A capillary column (HP 19091F-112, 25 m × 0.32 mm × 0.5 μm) was used with the following oven program: 80 °C, 1 min; 155 °C in 20 °C/min; 230 °C in 50 °C/min., constant for 3.5 min to separate the metabolites and helium as carrier gas. Concentration of the major SCFA (i.e. acetate, propionate and butyrate) was registered as referred to kilogram feces.

Quantitative evaluation of these metabolites was also inferred via investigation of proteins that are commonly recognised as being related to SCFA biosynthesis [33]. Here, abundances of proteins related to major SCFA were cumulatively considered to provide a quantitative estimation for each metabolite.

Results from direct and inferred estimation were standardised and subjected to Spearman correlation analysis by using the corrplot package of R v.3.1.2 software (http://www.R-project.org).

## Results

### 16S rRNA gene sequencing and metaproteome analysis revealed three adaptation periods

Sequencing of the V1-V2 region of the 16S rRNA gene produced 4.8 million reads (57,916 ± 2139 reads per sample). A Good’s coverage index greater than 98% showed sufficient sampling of our data and adequate depth. Reads were filtered and trimmed before being clustered into 3497 operative taxonomical units (OTUs) (Additional file 1: Table S1).

Adopted protocols for the metaproteomic investigation enabled a total of 9703 and 38,239 bacterial protein and peptide identification, respectively. Insights into the protein and peptide profile of each sample, as well as the respective abundance indexes for each of the identified entries are provided in Additional file 1: Table S2.

**Fig. 1:**
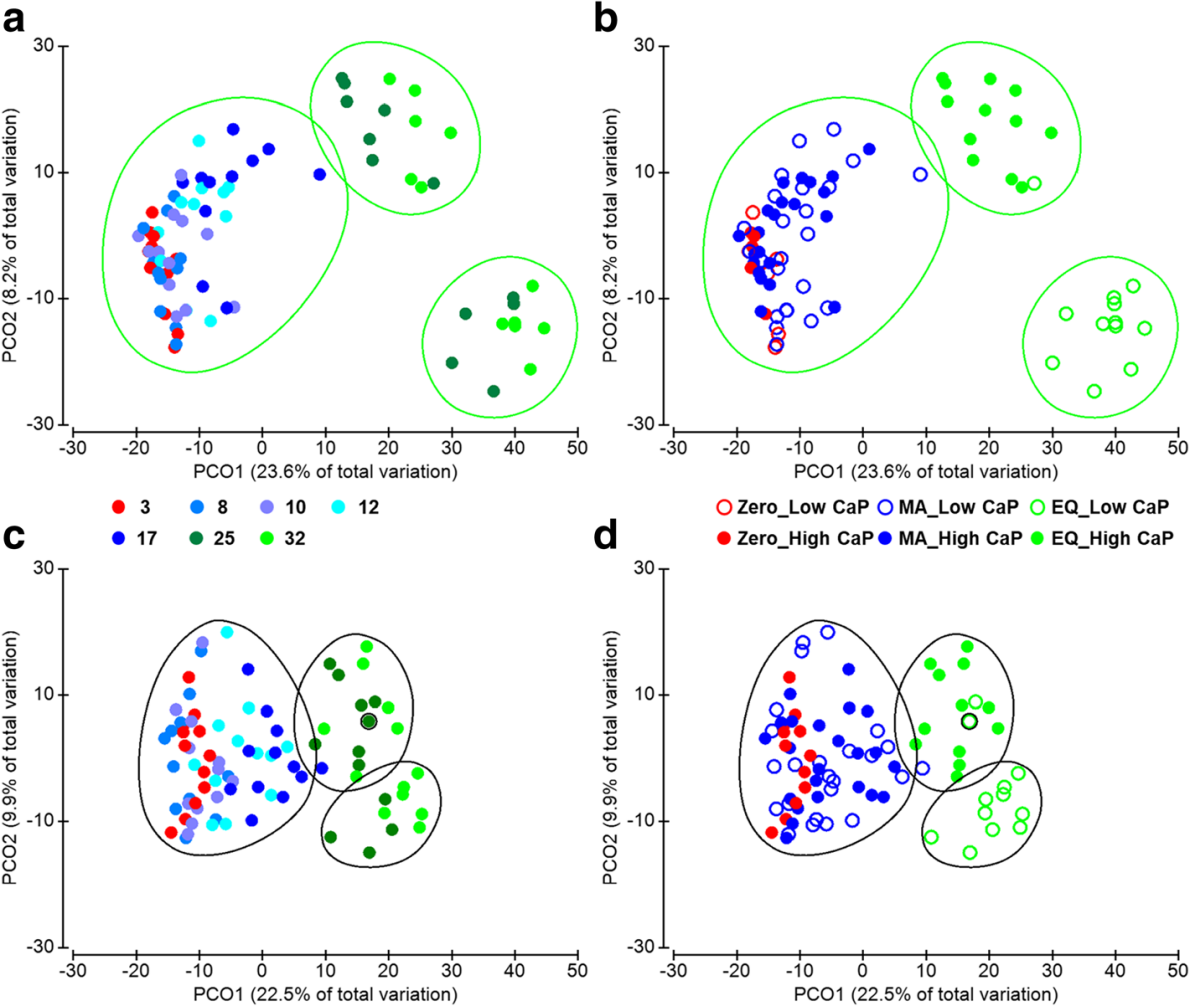
Samples ordination reveals three adaptation periods. **a** and **b** panels illustrate the ordination of the dataset obtained from the 16S rRNA gene sequencing approach. **c** and **d** panels show the metaproteomic dataset ordination. Datasets from both approaches are ordered on a sample basis. The time-dependent aggregation into three clusters is shown in panels **a** and **c**. Panel **b** and **d** include information in respect to the experimental diets. Similarity analysis showed 40% similarity in the sequencing dataset (green clusters) and 60% similarity in the metaproteomics dataset (grey clusters)

Both datasets, based on DNA and metaproteomic investigation, were depicted in a PCoA plot on a sample basis (Fig. 1). The samples ordination revealed a highly comparable clustering on a time point dependent manner along the PCO1 axis (Fig. 1a, c). Samples grouped into three clusters over the experimental time span (*p* = 0.001 for both datasets) suggesting that the adaptation process of the intestinal microbiota evolved throughout three main adaptation periods: zero (i.e. the phase prior to the experimental diet administration), metabolic adaptation (MA, adaptation period to the challenging diets) and equilibrium (EQ, last experimental period, where a new suited microbiota is established). The equilibrium achieved in the bacterial community at the EQ period is also supported by the PCO2 ordination where only EQ samples are further clustered according to the CaP supplementation of the experimental diets (*p* = 0.001) (Fig. 1b, d, Additional file 2: Figure S1C, D).

Identified OTUs were sorted according to the three adaption periods. This prior qualitative evaluation indicates the presence of a period-specific architecture of the microbiota featured by a gradual adaptation of the microbial communities, as suggested by the lower number of shared OTUs between the zero-EQ periods when compared to the zero-MA and MA-EQ pairs (Additional file 2: Figure S1A). Similarly, sorting of the protein dataset revealed that a variable number of proteins was uniquely identified in each of the three adaptation periods (1521, 595, 1927, respectively, for zero, MA and EQ period) whereas an equal number of proteins is shared between the MA-zero and MA-EQ periods. Only 13 proteins are shared between the zero and EQ periods supporting the achievement of a new homeostatic balance (Additional file 2: Figure S1B).

### Taxonomic distribution based on 16S rRNA gene sequencing and metaproteomics

OTUs with more than 10 associated reads appearing in more than one sample were selected to investigate the structure of the fecal microbiota. Taxonomic distribution based on the whole metaproteomic dataset did not provide noticeable shifts among the adaptation periods (Additional file 2: Figure S2), probably because of the presence of highly abundant housekeeping proteins. Thus, we focused on the unique proteins of each adaptation period to highlight the dynamics featuring the taxonomic composition of the fecal microbiota across the adaptation periods.

**Fig. 2:**
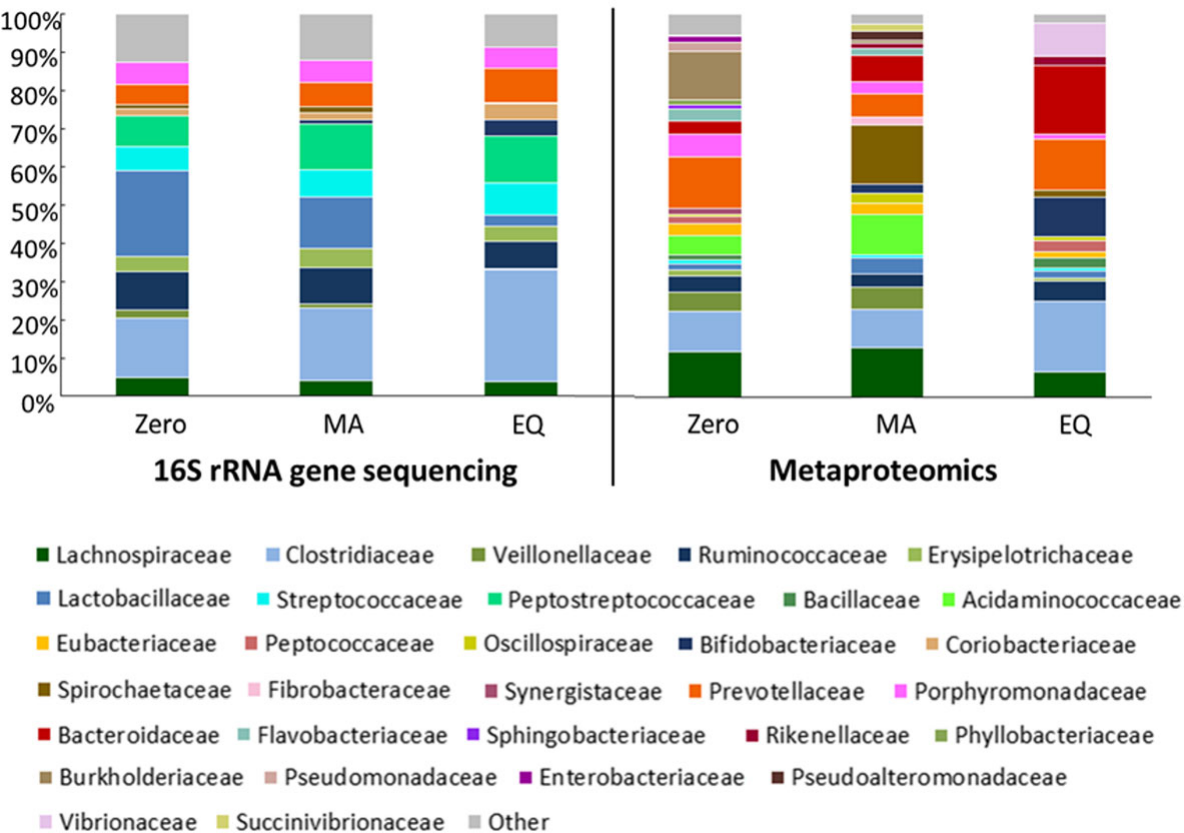
Gut microbiota composition changes in a time point-dependent manner. Bar chart displays the relative abundance of the bacterial families as assessed by 16S rRNA gene sequencing and label-free quantification metaproteomics. Both methods display a dynamic taxonomic composition among the adaptation periods. A higher taxonomic variability is visualized in the metaproteomic-based assessment when compared to the DNA-based approach

The DNA-based investigation revealed a dynamic composition of the fecal microbiota over the experimental time frame (Fig. 2) featured by an increased abundance of the Clostridiaceae and Prevotellaceae families in the EQ period (29.2 ± 2.21% and 8.9 ± 1.22%). The Peptostreptococcaceae increased in abundance with the administration of the experimental diets, showing a higher abundance in the MA (11.9 ± 0.74%) and EQ. (12.2 ± 1.42%) periods. Similarly, Bifidobacteriaceae showed a time-dependent increase in abundance. At the zero period, the abundance of Bifidobacteriaceae sequences were 0.1% ± 0.06, whereas in the EQ period this family showed an abundance of 4.3% ± 1.07. Contrarily, the gut microbiota re-structuration triggered by the challenging diets showed a gradual decrease of the family Lactobacillaceae from 22.4 ± 1.76% of abundance observed in zero period to 3 ± 0.52% in the EQ period (Fig. 2).

The metaproteomic investigation confirmed the restructuring of the fecal microbiota architecture on a time point dependent manner. Compared to the DNA-based approach, a higher bacterial heterogeneity is shown in the description of the active bacterial community. The abundance of proteins affiliated to Clostridiaceae and Bifidobacteriaceae family reflects the observed results at the 16S rRNA gene level. Proteins of Clostridiaceae (10.5 ± 1.0% at the zero period to 18.6 ± 1.2% of the total protein abundance scored in the EQ period) and Bifidobacteriaceae (0, 2.4 ± 0.03% and 10.2 ± 0.7% in zero, MA and EQ periods respectively) increased gradually within time (Fig. 2).

Proteins affiliated to Erysipelotrichaceae indicate a reduced abundance in the EQ period (respectively 1.6 ± 0.6% and 0.8 ± 0.01% of protein abundance in the zero and EQ period). None of the proteins related to Peptostreptococcaceae family passed the filters and thresholds applied to the dataset, leading to its exclusion from the taxonomic assessment of the active bacterial community.

Proteins associated with Bacteroidaceae strongly increase in abundance with time. The reverse trend is shown for Lachnospiraceae and Veillonellaceae members, whose protein abundances are firmly reduced in the EQ period. Similarly, the abundance of proteins affiliated to Burkholderiaceae is progressively reduced during the MA period until the EQ, where it was not detectable with the investigation method (Fig. 2).

The abundance of proteins related to Prevotellaceae members collapse during the MA period and are restored in the EQ period, suggesting Prevotellaceae as a bacterial family involved in important housekeeping functions carried out in both homeostatic balances (zero and EQ). The opposite effect was observed for Lactobacillaceae, Spirochaetaceae and Acidaminococcaceae members, whose protein repertoire is highly pronounced in the MA period, indicating these families are potentially involved in driving the shift from the zero to the EQ microbiota (Fig. 2).

### Functional adaptation of the intestinal microbiota

Out of the total protein repertoire, the “unique proteins” for each of the three adaptation periods (Additional file 2: Figure S1B), were considered for a functional categorization in order to investigate the overtime functional shift of the fecal microbiota. Here, shared proteins were excluded since they are most likely involved in the highly conserved housekeeping functions taking place in all the adaptation periods. Moreover, shared proteins account for most of the total protein abundance (Additional file 2: Figure S3), therefore, their consideration hinders a clear visualisation of the hypothesised gradual functional shift of the microbiota (Additional file 2: Figure S4), as also supported by statistical results (i.e. three adaptation periods, *p* = 0.001).

The LFQ values of the sorted proteins in each adaptation period were compared to each other to identify the major proteins responsible for the observed statistical differences. Only proteins scoring at least 5-fold changes between adaptation periods were considered for a further functional classification into KEGG biochemical pathways. Global representation of the screened proteins according to their LFQ ratio, as well as their functional categorization into KEGG biochemical pathways is provided in Additional file 2: Figures S5 and S6.

Functional profiles drawn for the three adaptation periods clearly show a dynamic change of the microbiota, as supported by the fluctuating expression levels of diverse pathways among the adaptation periods as well as the emergence of new, other paths in the MA and EQ periods (Additional file 2: Figure S6).

**Fig. 3:**
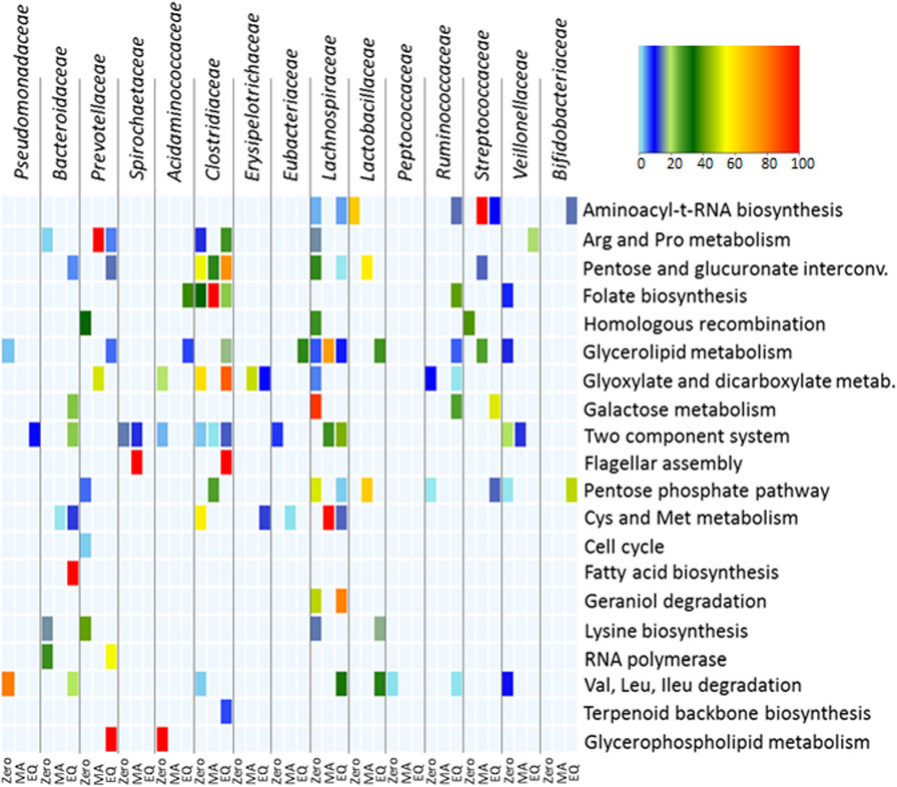
Microbiota members are involved in a variety of diverse biochemical pathways. The heat map shows the contribution of the top 10 most abundant bacterial families in the selected pathways in each adaptation period. Bacterial families exhibit specific involvement in the biochemical pathways, with a contribution that changes in dependence of the considered bacterial family and adaptation period

The heat map shown in Fig. 3 summarises the biochemical pathways with the highest variability in abundance between the adaptation periods including along with the bacterial families that contributed to their expression.

**Fig. 4:**
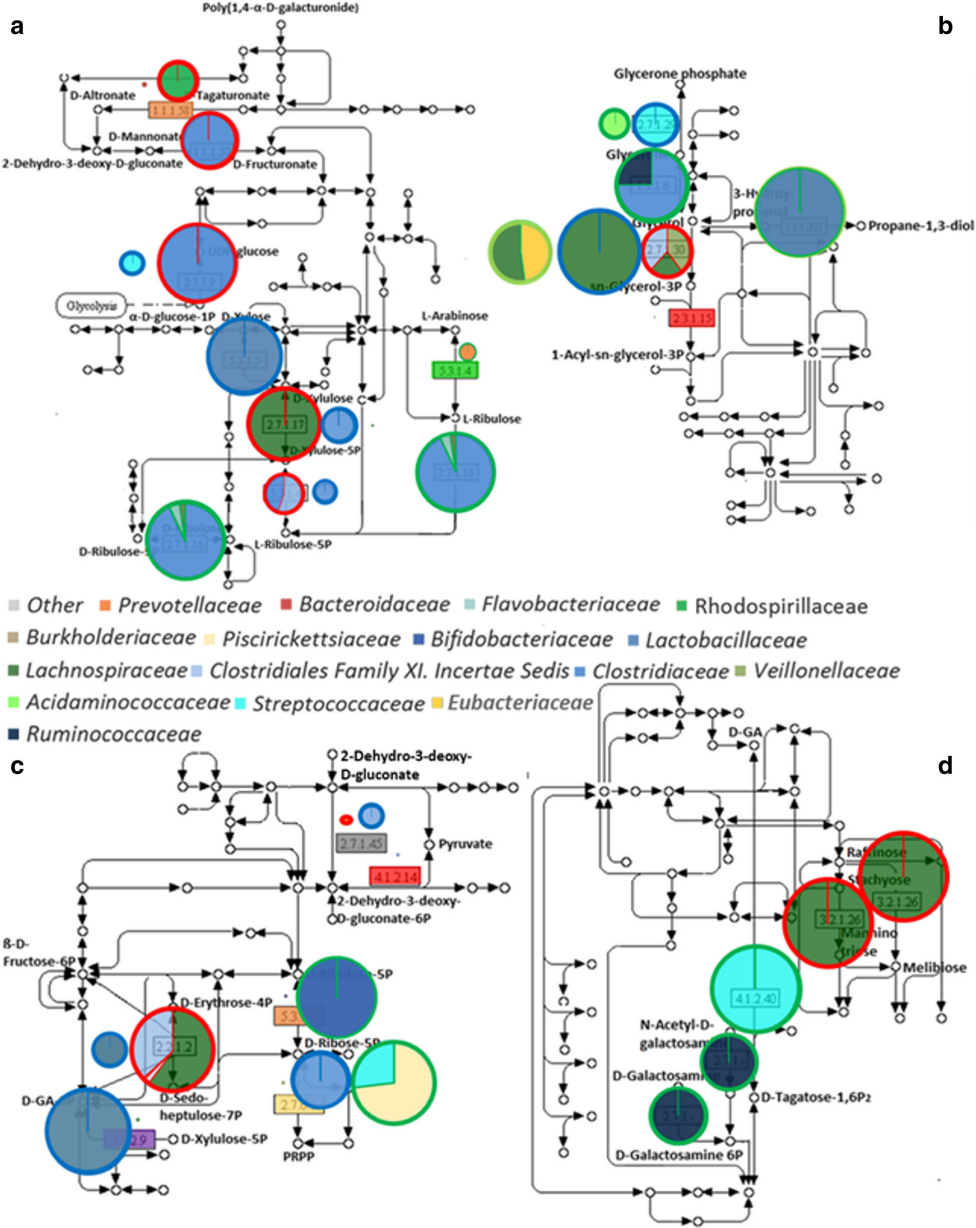
Microbial community exhibit a varying concern in selected carbohydrates pathways. Panel **a** Pentose and glucuronate interconversions, **b** glycerolipid metabolism, **c** pentose phosphate pathway and **d** galactose metabolism. Differently colored fillings of the pies indicate the bacterial families involved in the production of each of the identified proteins. Pie size is representative of the protein abundance, normalised on a time point basis. This normalisation highlights the portion of path of major concern for each of the three adaptation periods. The color code of the pie framing stands for *red*: zero, *blue*: MA and *green*: EQ. Colored squares, indicating the identified proteins, are used when pies contour does not allow for a clear distinction between the adaptation periods due to the reduced pie size. *Red*: zero; *blue*: MA; *green*: EQ; *yellow*: all periods; *grey*: zero/MA shared; *orange*: Zzro/EQ shared; *purple*: MA/EQ shared

The Clostridiaceae family, whose abundance increases in the EQ period, showed a concomitant increase in abundance in some of the selected pathways such as the pentose and glucuronate interconversion pathway (ko00040), and the glyoxylate and dicarboxylate metabolism (ko00630). Proteins of Bifidobacteriaceae members showed only low to medium abundance in the aminoacyl-t-RNA biosynthesis (ko00970) and the pentose phosphate pathway (ko00030) once achieving the new equilibrium (EQ period). This evidence suggests that the gradual increase of the Bifidobacteriaceae registered in the metaproteomic-based phylogenetic taxonomic assessment reflected a bacterial activity concerned in other aspects of the functional adaptation of the gut bacterial community. The reduced abundance of the family of Lachnospiraceae is functionally reflected by its sudden drop in the galactose metabolism (ko00052) and glycerolipid metabolism (ko00561). Similarly, the reduced abundance of Veillonellaceae results in a decreased abundance in the glycerolipid metabolism and pentose phosphate pathway. The increased abundance of Lactobacillaceae members in the MA period is accordingly related to a boosted number of proteins in the pentose, glucuronate interconversion and pentose phosphate pathway (Figs. 3 and 4).

A further focus on the carbohydrate metabolic pathways and the related bacterial families is summarised in Fig. 4 and Additional file 2: Figure S6. In accordance with the previous evidence, a diverse fraction of the bacterial community is concerned in the carbohydrate metabolism in each of the adaptation periods. Furthermore, a detailed investigation reveals quantitative differences in the portion of the major biochemical pathways for each of the adaptation periods, underlining a different impact of every adaptation period on the selected carbohydrate pathways. In the pentose and glucuronate interconversion KEGG pathway (Fig. 4a), proteins of the zero samples showed high abundances throughout the whole path, highlighting that zero bacterial community is mainly focused on facing complex substrates and improving the carbon and energy uptake. Proteins affiliated to glycerolipid metabolism indicate zero and MA samples as being concerned in the biosynthesis of triglycerides and glycerolipids. A similar functional profile was also identified in EQ samples along with their strong implication in using glycerol as a carbon and energetic source (Fig. 4b).

Investigation of proteins of the pentose phosphate pathway highlighted that diverse bacterial proteins are involved in common functions (Fig. 4c). From a quantitative point of view, the identified protein repertoire showed a higher efficiency of the EQ-related bacterial community in the production of intermediates entering the glycolytic route when compared to the other adaptation periods. Nevertheless, a similar function is also achieved by the MA-related microbiota using a different route within the same biochemical pathway.

Proteins categorised into the galactose metabolism KEGG pathway showed a major abundance in the zero and EQ samples (Fig. 4d). The bacterial community of the zero samples, in line with the previous observation (Fig. 4a), is almost exclusively concerned in widening the array of substrates through the production of more easily digestible metabolites. The EQ-related bacterial community, in contrast, is involved in the production of N-acetylgalactosamine-specific component IIA (EC 2.7.1.-) and tagatose 1,6-diphosphate aldolase (EC 4.2.1.40). The first is a component of the phosphotransferase system, one of the major bacterial mechanisms for the uptake of complex sugars whereas the latter enzyme is a class I aldolase also involved in essential metabolic pathways such as gluconeogenesis and glycolysis [34, 35].

The protein dataset was also analysed for the presence of glycosyl hydrolases (GH) and glycosyl transferases (GT) via the CAZy database [36] (Additional file 1: Table S3). In general, qualitative identification of the GH and GT families is not changing between the adaptation periods. Enzymes of the families GH13 and GH36 are more abundant in the EQ samples, indicating a higher concern of the EQ bacterial community in the hydrolysis of the alpha-bond of glycosylated macromolecules (glycolipids, glycoproteins) and large polysaccharides (starch and glycogen) when compared to the zero counterpart. In contrast, zero samples exhibited a higher abundance of enzymes affiliated to GH1, GH43, GH3 and GH95 families enabling a broad range of functions [36]. In line with the taxonomic results, Prevotellaceae and Lachnospiraceae were among the major producers of the GHs identified in the zero samples, whereas Prevotellaceae, Clostridiaceae and Bacteroidaceae were found to be some of the main contributors to the GHs of the EQ-related bacterial community (Additional file 1: Table S3). The abundance of proteins related to the GT5 family is higher in zero samples, indicating a strong concern of the microbiota in the formation of the alpha-1,4-bonds required in the biosynthesis of polysaccharides such as glycogen and starch. Their production is needed in enteric bacteria to ensure a rapid growth in the intestinal environment where there is high competition and occasional lack of nutrients [37]. An opposite trend is observed in the EQ bacterial community, showing a higher abundance of enzymes affiliated to the GT35 family, whose main function is the phosphorylation-mediated degradation of starch and glycogen [36]. Similarly to GHs, the identified bacterial families showed a different contribution to the production of the GTs depending on the adaptation period. Lachnospiraceae members produced a higher abundance of GT5 in zero samples when compared to the EQ counterpart. Bifidobacteriaceae and Bacteroidaceae, in contrast, are strongly involved in the EQ-related GTs and did not show participation in the production of GTs of the zero samples. Equal contributions of Prevotellaceae members are observed in the production of the GTs in all the adaptation periods (Additional file 1: Table S3).

### Short-chain fatty acids biosynthesis

The whole metaproteomic datasets of the zero, MA and EQ periods were checked for the presence of proteins which are indicators of SCFA production as previously reported [33] and listed in the legend of Additional file 2: Figure S7. Investigated enzymes are involved in the biosynthesis of formate, acetate, propionate and butyrate. A minor part of the whole dataset concerned the SCFA indicators of our choice, corresponding to 3.7 ± 0.1%, 2.8 ± 0.1% and 3.0 ± 0.09% of the total LFQ of zero, MA and the EQ period, respectively. The three adaptation periods accordingly indicated proteins involved in the propionate synthesis pathway as the most abundant followed by proteins of the butyrate, acetate and formate production pathways. The abundance of the butyrate-producing enzymes was not changing over time, whereas the abundance of the propionate indicators showed a gradual decrease, counterbalanced by the progressive increase in the abundance of acetate and formate-producing enzymes (Additional file 2: Figure S7).

Details on the bacterial specimen involved in the SCFA production are provided in Additional file 2: Figure S8. Results of the GC measurements of SCFA showed acetate as the most abundant SCFA followed by propionate and butyrate with an average abundance of 59 ± 3.3, 22.6 ± 1.1 and 16.2 ± 1.1 mmol/kg feces regardless the experimental time points (Additional file 1: Table S4 and Additional file 2: Figures S7 and S8). Similar to the metaproteomics outcomes, no notable overtime changes were observed for butyrate concentration, but a dietary effect is observed with an increase of butyrate in all samples from diets with low CaP levels. Propionate was registered with an increased amount in EQ samples (24.4 mmol/kg feces) when compared to the zero counterpart (20.9 mmol/kg feces) but no dietary effect was observed. No gradual changes could be shown for acetate but diets with low CaP levels exhibited a lower acetate concentration at day 32 (Additional file 1: Table S4 and Additional file 2: Figures S7 and S8). Correlation of the metabolite measurements with the protein abundances revealed a scarce correlation between the results, with a Pearson correlation coefficient either positive or negative close to zero (ranging from − 0.33 to + 0.13) (Additional file 2: Figure S9).

### Host proteome is affected by changes in the intestinal microbiota

**Fig. 5:**
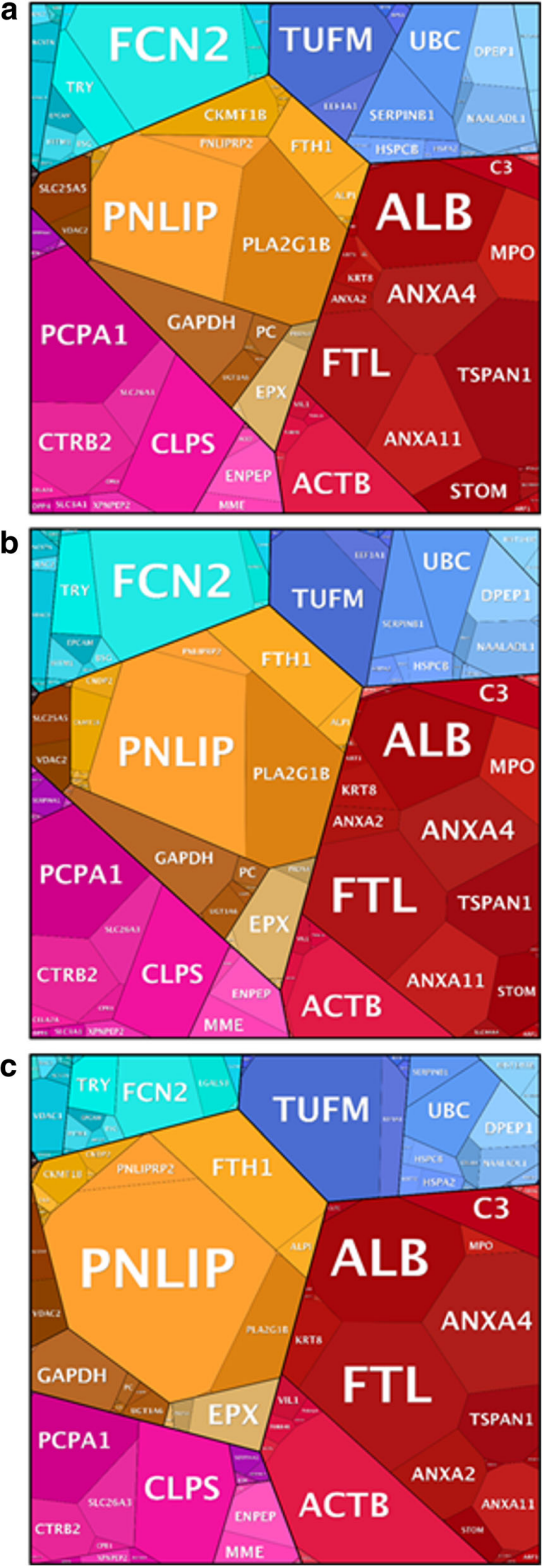
Host proteome changes along with the remodelling of its gut metaproteome. Voronoi diagrams show the host proteome of zero (**a**), MA (**b**) and EQ (**c**) samples. Identified proteins are visualised as polygons, whose area reflect their relative abundance. Gene IDs are detailed for each polygon

In this study, a total of 513 pig proteins were identified and functionally categorised into proteomaps according to the adaptation periods described above [38]. A quantitative representation of the ongoing host functions (visualized as gene names (GN)) over the experimental time span is shown in Fig. 5.

In general, abundance profiles of the animal proteins confirmed the previously observed gradual adaptation process highlighting two distinct representations for zero and EQ samples, whereas, MA period samples recorded intermediate abundance values (*p* = 0.001, Fig. 5).

Abundance of proteins identified in the zero period depicted a strong implication of the host in cell growth, motility and cell cycle, as supported by the high abundance of proteins such as actin alpha1 (GN = ACTA1) and annexin A4 (GN = ANXA4). The functional profile assessed in the MA period showed common functions to the two equilibrium conditions (zero and EQ). However, the higher abundance of proteins such as phospholipase A2 (GN = PLA2G1B) in MA samples suggests a host effort in preserving the functional homeostasis of the gastric mucosa by monitoring the structure of its microbial community. Similarly to the zero period, EQ samples were also involved in cell motility and cell cycle. However, the higher abundance of tyrosine 3-monooxygenase/tryptophan 5-monooxygenase activation protein zeta (GN = YWHAZ), annexin A11 (GN = ANXA11) and tubulin beta 4B class IVb (gene name TUBB4B), involved in mitotic cell cycle, cell division and cytoskeletal organization respectively, leads us to the assumption that there is an important concern of the host in organ enlargement and animal growth at the EQ period.

## Discussion

An improved knowledge about the intestinal microbiota of pigs is of interest for translational research, animal husbandry optimisation and animal health improvement. Kim et al. [39] described the natural, age-dependent shift of the fecal microbiota composition of commercial swine, emphasizing the importance of animal’s age as a factor shaping the pigs intestinal microbiota. The same study also determined the trustworthiness of results obtained from pig groups rather than results arising from the same trial conducted at the level of individual animals [39]. The current study investigated the gradual adaptation mechanisms of the pigs’ gut bacterial community during a shift of experimental diets differing protein sources and levels of CaP. The statistical analyses of both DNA- and protein-based datasets showed a clustering of the investigated samples overtime, revealing a gradual adaptation of the fecal microbiota to the experimental diets. The microbiota adaptation process was hypothesised throughout three main periods; the first of which (i.e. zero) represents the unaltered gut microbial community prior to the administration of the experimental diets. The second period (i.e. MA) describes the structural and functional transition of the fecal microbiota in the attempt to face the challenging factor. The third period (i.e. EQ) depicts the newly established equilibrium of the gut microbial community, as supported by further clustering of the samples according to the diverse levels of CaP administered to the diets. Previous studies on the intestinal microbiota of animals kept at diets with high CaP levels reported an increased amount of *Prevotella* spp. along with other Enterobacteriaceae and Clostridiaceae members [9, 14]. At the stomach level, high CaP levels were associated with an increased amount of Lactobacillaceae and a reduced portion of *Prevotella* and *Streptococcus* [14]. A further study concluded that the overall bacterial community rather than specific groups is affected by feeding diets with varying levels of CaP [40]. This could be observed in the present study where an overall remodelling of the bacterial community was observed without identifying specific factors, like OTUs or proteins, which may cause this effect. We believe that the fecal microbiota is shaped by the changing CaP levels through a multitude of ways, ranging from the modified physicochemical environment to altered relationships between microorganisms and the host. Here, we measured a decreased amount of SCFA, especially acetate, at low CaP levels but an enhanced concentration of butyrate (Additional file 1: Table S4). This could indicate a functional shift with beneficial effects for the host as butyrate serves as an energy source for the colonic epithelium [41]. In broilers, it was demonstrated that the stress induced by a reduced CaP supplementation is subsequently mirrored in the gastrointestinal tract-related microbial community [13]. As no increase of stress-related proteins were found during the present study, the change in the microbial community was probably caused by an altered metabolism of the host linked to a modified secretion of host metabolites into the gut lumen. Thus, further investigations are required to define specific factors involved in the CaP-dependent alteration of the intestinal microbiota. In contrast to CaP, no effect imputable to the diverse protein sources over the experimental time frame was identified. This is probably due to a large fraction of corn (33–67%) in both diet formulations, which masks the possible effect of the soybean meal and field peas supplementation, as already highlighted in other studies [42, 43]. In contrast, Rist et al. observed a shift in the intestinal microbiota composition due to increased dietary corn supplementation [44].

The structure of the fecal microbiota was investigated through 16S rRNA gene sequencing and metaproteomic analyses. Changes in the abundance of some bacterial families such as Clostridiaceae, Bifidobacteriaceae and Lactobacillaceae are detected by both investigation strategies indicating a parallel structural and functional remodelling of the gut bacterial community. Even though, a diverse general microbiota composition is drawn by the two adopted approaches.

The protein-based microbiota assessment described a very dynamic structure of the bacterial community, highlighting the disappearance of some bacterial families and the presence of new ones along the complete experimental time span. The emergence of new bacterial families and the strong changes of functions observed during the three adaptation periods are a clear example of how the process of microbiota re-structuration occurs overtime and how the diverse bacterial entities synergistically co-operate to form a balanced microbial community. This enables a better facing of the challenging diets and adaptation to the new surrounding environment. Compared to the DNA-based investigation, the metaproteomic-based taxonomic assessment identified a higher bacterial heterogeneity at both family and phyla level. The reported divergence of results is most likely imputable to the different principles these methods are based on. Both methods target different biological macromolecules and thus, are destined to diverse technical issues [12]. Moreover, we retain that metaproteomics enables the identification of a higher bacterial complexity since the changes in the abundance of expressed proteins are detected earlier than changes in the number of the DNA copies targeted by 16S rRNA gene sequencing. Similar evidence was observed in previous investigations. Tang and colleagues highlighted inconsistencies between the DNA and protein-based assessment of the microbiota composition [45]. Moreover, other studies described a higher bacterial complexity in metaproteomic datasets than in 16S rRNA gene sequencing data [12, 46].

Uniquely identified proteins for each of the three adaptation periods were subjected to functional classification. A functional classification of the whole metaproteome has been attempted, but a clear description of the gradual functional shift of the gut bacterial community was not possible. A plausible reason for this is that the shared proteins are involved in housekeeping functions, thus their consideration masks the statistically predicted gradual shift of the fecal microbiota. Moreover, the abundance of shared proteins counts for the most of the total LFQ indexes of each adaptation period, hampering the masking effect arising from the consideration of the shared proteins.

Functional profiles of the bacterial communities in the diverse adaptation periods reveal a dynamic change of the bacterial activity. In line with the taxonomic assessment, the bacterial families responsible for a phase-specific architecture of the fecal microbiota are also among those families active in the biochemical pathways causing the diverse functional profiles of each adaptation period. We focused our attention on the biochemical pathways showing the highest abundance variability is the major factor responsible for the phase-specific functional profiles of the fecal microbiota. Interestingly, almost all the changing bacterial families highlighted in the taxonomic assessment of the fecal microbiota are involved in carbohydrate-related pathways such as pentose and glucuronate interconversion, glycerolipid metabolism, pentose phosphate pathway and galactose metabolism. Focusing on these pathways highlighted that for each adaptation period, different reactions of the paths are concerned in a quantitatively different manner. Therefore, even though the diverse bacterial communities appeared to be involved in common pathways, internal investigation of the paths revealed a diverse array of functions performed by the bacterial community depending on the adaptation periods, thus the variety of systems enrolled to achieve convergence points (for example, entering the glycolysis).

Based on the identified protein repertoire and their functional categorization, we speculate that the zero period represents a thriving bacterial community whose composition and functional equilibrium have not been altered by external factors. This enables a deep specialization of the bacterial community, as supported by the high abundance of phosphoribulose isomerase (EC 5.1.3.4), tagaturonate reductase (EC 1.1.1.58), beta-fructofuranosidase (EC 3.2.1.26) and mannonate oxidoreductase (EC 1.1.1.57) suggesting a strong involvement of the zero bacterial community in facilitating sugar uptake and digestion [47]. In addition, this enlarges the substrate array to maximise feed conversion, by improving carbon and energy uptake [48, 49].

The EQ period in contrast, describes a bacterial community in a stage of freshly achieved homeostasis, thus still refining its functional profile for a better adaptation to the surrounding environment. Functions related to the widening of the substrates array and facing complex carbohydrates are still expressed, but at a lower level than observed in zero microbiota. Nevertheless, the high abundance of the glycerol dehydrogenase (EC 1.1.1.6) and 1,3-propanediol dehydrogenase (EC 1.1.1.202) observed in the glycerolipid metabolism KEGG pathway reveals a possible implication of the EQ bacterial community in alternative strategies to improve carbon and energy yield through the use of glycerol as a carbon and energetic source [50, 51].

On the other hand, the increased abundance of phosphoriboisomerase (EC 5.3.1.6) and tagatose 1,6-diphosphate aldolase (EC 4.2.1.40) suggests a higher concern of the EQ-related bacterial community in entering the glycolytic route [52] in order to yield the energy required to complete the specialization process for an optimal settlement in the new host environment.

Investigation of the MA period proteins describes a transitory bacterial community featured by intermediate evidence in terms of both composition and function. Here, the overtime increase of the abundance of the enzyme ribose-phosphate diphosphokinase (EC 2.7.6.1) suggests an increasing ability of the bacterial community of numerous biosynthetic processes, such as the de novo biosynthesis of purines and pyrimidines [53].

Indicators of SCFA production were sorted out of the total metaproteomic dataset, in order to infer the SCFA production in the different adaptation periods. Correlation analysis of the predicted SCFA production with the direct measurements of the metabolites indicated a high correlation coefficient for acetate exclusively. The scarce correlation scored for all other metabolites is probably due to the fact that bacteria can produce SCFAs through a variety of metabolic routes, each of which is featured by a diverse array of enzymes [54]. Based on this finding, we believe that only acetate was produced through the route targeted by the indicators of our choice; whereas the other metabolites were produced through metabolic routes (i.e. enzymes) that were not identified by the set of indicators used in our investigation.

Alteration of the intestinal microbiota, as well as its gradual adaptation, is also reflected in the host proteome. Protein profile of the zero and MA samples showed a strong participation of the host in shaping the intestinal microbiota composition for a better facing of the new diets. EQ samples instead are involved in host cell division and organ enlargement. In this regard, we retain that the freshly assessed bacterial community built an optimal growth environment by providing nutrients and energy to its host. This determines an increased tendency in intestine enlargement in EQ samples rather than zero ones. However, care should be taken when comparing the growth capability of zero and EQ samples since these samples do not belong to animals of the same age. Therefore, some of the variability observed in their protein profile could be age-related and not exclusively due to the intestinal microbiota changes. Moreover, the sample preparation protocols applied in the current study preferentially target bacterial proteins, resulting in a lower coverage of the host proteome that does not allow for a deep and complete investigation of the complex interaction network established between the intestinal microbiota and its host.

## Conclusion

For the first time, this study presents insights into the gradual adaptation of the porcine intestinal microbiota challenged by experimental diets. Taxonomic and functional dynamics of the bacterial community have been depicted through 16S rRNA gene sequencing and metaproteomics until the achievement of a stable bacterial community. Besides the dynamic changes of the microbiota, this study defines the duration of the metabolic adaptation process required by the intestinal microbiota. This is of a great importance for the design of future nutritional studies. Moreover, considering the high similarities between the porcine and human gastrointestinal tract anatomy and physiology, the findings of the current study might imply in the design of human-related nutritional studies as well as the characterization of the human intestinal microbiota when challenged by the alteration of external factors such as the diet. Nevertheless, this study focused on the investigation of the major changes of the fecal microbiota, therefore further complementary studies investigating other structural and functional aspects of the challenged microbial community are desirable.

## Additional files

Additional file 1: Table S1. Table report the fast sequence of each OTU. **Table S2.** Metaproteomic dataset. A) Table report a summary of information on the peptides/proteins identified. B) Table include further insights on peptide identification and their implication in protein IDs inference. **Table S3.** Glycosyl hydrolase and glycosyl transferase production. A) Panel reports the identified GHs and GTs. The relative abundance (%) is detailed for each GH and GT family in each adaptation period. B) Panel shows the relative contribution (%) of the bacterial specimens encoding for the major GH and GT families identified over the three adaptation periods. C) Panel report the list of proteins classified in each of the identified GH and GT, along with the LFQ index and the relative bacterial families. **Table S4.** SCFA concentration. Table of the total SCFA, acetate, propionate and butyrate concentration of all animals at days 3 and 32. (ZIP 108295 kb)

Additional file 2: Figure S1. Venn diagrams display the number of OTUs (A) and proteins (C) attributed to the three adaptation periods. B, D are tables showing the respective *p* values calculated by a pair-wise comparison to show the significant differences between the time points and diets. **Figure S2.** Taxonomic assessment of the samples at each of the selected experimental time points (days). The entire metaproteomic dataset (i.e. both unique and shared proteins) is considered for the fecal microbiota taxonomic assessment. **Figure S3.** LFQ distribution among the adaptation periods. Pie charts represent the relative distribution of the abundance index of the proteins identified in zero (A) MA (B) and EQ (C) samples. **Figure S4.** Functional classification of the identified proteins by their categorization into COG classes (A) and KEGG biochemical pathways (B). Only categories with a cumulative abundance higher that 1% of the total LFQ abundance index are included in the visualisation. A functional classification of the samples at all the selected experimental time points (days) is provided. **Figure S5.** Heat map displays a list of proteins whose abundance ratio is changing between adaptation periods of at least 5-fold. Abundance indexes of each protein in the diverse adaptation periods are shown as log LFQ. **Figure S6.** Protein classification into KEGG biochemical pathways. Abundance of the pathways is expressed as a relative percentage for each of the adaptation periods. The only pathways scoring at least 2.5-fold change between the adaptation periods are visualised. **Figure S7.** SCFA production as assessed through the metaproteomic (A and B) and conventional approach (C and D). A Abundance of the enzymes, selected as indicators of SCFA production, out of the total LFQ abundance indexes. B Distribution of the indicators for the major SCFA production, across the diverse adaptation periods. C Summary of the SCFA measurements in the zero and EQ period, on an animal basis. D Relative production of the major SCFA as assessed through GC measurement. The proteins involved in the prediction of the SCFAs production are formate production: COG1882. Acetate production: COG0282; COG0280; COG1012. Propionate production: COG0777, COG4799; COG2185, COG1884; COG4577. Butyrate production: COG4770; COG0183; COG1028, COG1064; COG3426; COG1250, COG1024. **Figure S8.** SCFA production by gut microbial commensals. In the metaproteomic approach, the SCFA production has been inferred through investigation of the quantitative expression of enzymes involved in SCFA biosynthesis. A Formate production: COG1882. B Acetate production: COG0282; COG0280; COG1012. C Propionate production: COG0777, COG4799; COG2185, COG1884; COG4577. D Butyrate production: COG4770; COG0183; COG1028, COG1064; COG3426; COG1250, COG1024. **Figure S9.** A Correlogram displays the relationships occurring between the investigation approaches, as well as the relationships between metabolites production as measured according to either metaproteomics or the conventional GC-based approach. B The correlation coefficient for each of the compared pair is also provided. (ZIP 1560 kb)
