# Supplementary material for: Dietary changes in nutritional studies shape the structural and functional composition of the pigs’ fecal microbiome—from days to weeks
Source: Microbiome. 2017 Oct 27;5:144. doi: 10.1186/s40168-017-0362-7 (PMC5659009; doi:10.1186/s40168-017-0362-7)
Supplement: Supplementary file 2 — Venn diagrams display the number of OTUs (A) and proteins (C) attributed to the three adaptation periods. B, D are tables showing the respective p values calculated by a pair-wise comparison to show the significant differences between the time points and diets. Figure S2. Taxonomic assessment of the samples at each of the selected experimental time points (days). The entire metaproteomic dataset (i.e. both unique and shared proteins) is considered for the fecal microbiota taxonomic assessment. Figure S3. LFQ distribution among the adaptation periods. Pie charts represent the relative distribution of the abundance index of the proteins identified in zero (A) MA (B) and EQ (C) samples. Figure S4. Functional classification of the identified proteins by their categorization into COG classes (A) and KEGG biochemical pathways (B). Only categories with a cumulative abundance higher that 1% of the total LFQ abundance index are included in the visualisation. A functional classification of the samples at all the selected experimental time points (days) is provided. Figure S5. Heat map displays a list of proteins whose abundance ratio is changing between adaptation periods of at least 5-fold. Abundance indexes of each protein in the diverse adaptation periods are shown as log LFQ. Figure S6. Protein classification into KEGG biochemical pathways. Abundance of the pathways is expressed as a relative percentage for each of the adaptation periods. The only pathways scoring at least 2.5-fold change between the adaptation periods are visualised. Figure S7. SCFA production as assessed through the metaproteomic (A and B) and conventional approach (C and D). A Abundance of the enzymes, selected as indicators of SCFA production, out of the total LFQ abundance indexes. B Distribution of the indicators for the major SCFA production, across the diverse adaptation periods. C Summary of the SCFA measurements in the zero and EQ period, on an animal basis. D Relative production of the major SC [file 40168_2017_362_MOESM2_ESM.zip › Figure S9_ Correlogram.pdf]

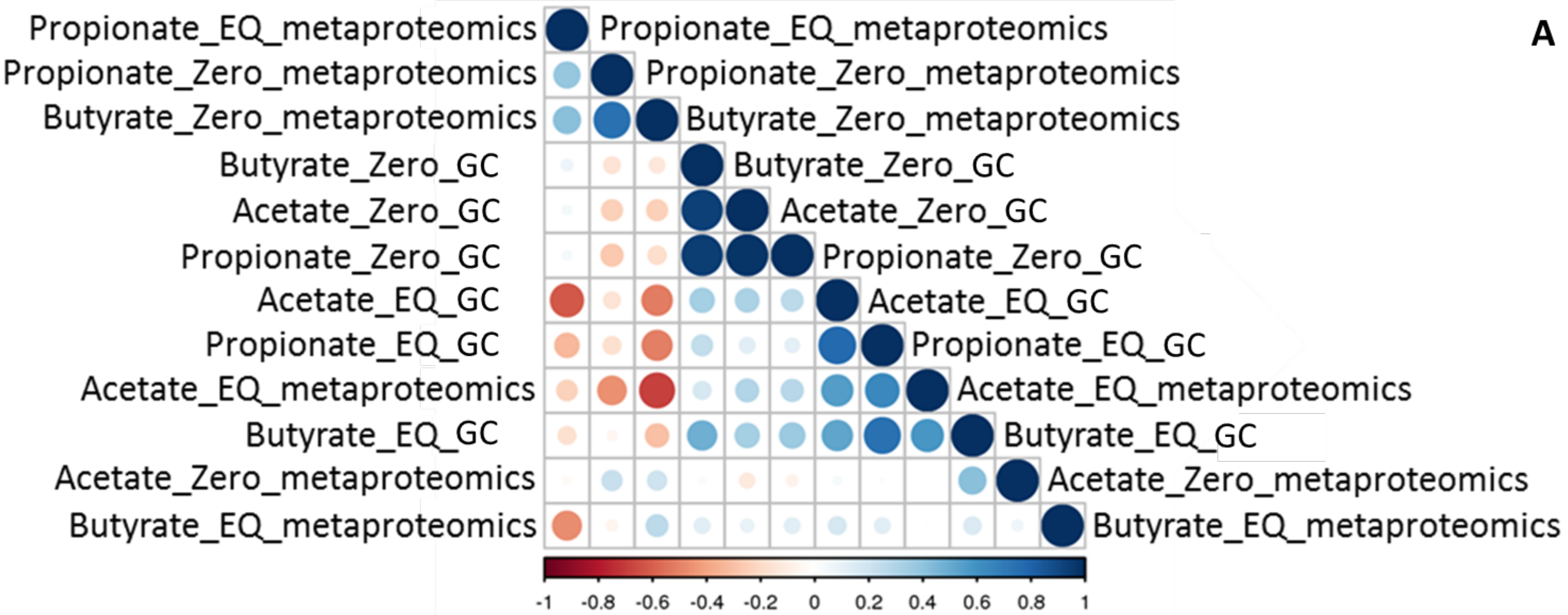

B

Propionate_EQ_metaproteomics	1	Propionate_EQ_metaproteomics											
Propionate_Zero_metaproteomics	0.38	1	Propionate_Zero_metaproteomics										
Butyrate_Zero_metaproteomics	0.41	0.74	1	Butyrate_Zero_metaproteomics									
Butyrate_Zero_GC	0.07	-0.15	-0.13	1	Butyrate_Zero_GC								
Acetate_Zero_GC	0.04	-0.24	-0.24	0.93	1	Acetate_Zero_GC							
Propionate_Zero_GC	0.04	-0.27	-0.18	0.94	0.97	1	Propionate_Zero_GC						
Acetate_EQ_GC	-0.62	-0.15	-0.52	0.33	0.31	0.26	1	Acetate_EQ_GC					
Propionate_EQ_GC	-0.33	-0.17	-0.51	0.24	0.13	0.11	0.77	1	Propionate_EQ_GC				
Acetate_EQ_metaproteomics	-0.23	-0.46	-0.69	0.17	0.29	0.27	0.55	0.64	1	Acetate_EQ_metaproteomics			
Butyrate_EQ_GC	-0.17	-0.05	-0.3	0.48	0.33	0.36	0.52	0.74	0.58	1	Butyrate_EQ_GC		
Acetate_Zero_metaproteomics	-0.03	0.22	0.2	0.02	-0.12	-0.07	0.03	0.01	-0.01	0.41	1	Acetate_Zero_metaproteomics	
Butyrate_EQ_metaproteomics	-0.47	-0.06	0.26	0.13	0.09	0.13	0.17	0.13	-0.01	0.16	0.06	1	Butyrate_EQ_metaproteomics
