# Supplementary material for: Dietary changes in nutritional studies shape the structural and functional composition of the pigs’ fecal microbiome—from days to weeks
Source: Microbiome. 2017 Oct 27;5:144. doi: 10.1186/s40168-017-0362-7 (PMC5659009; doi:10.1186/s40168-017-0362-7)
Supplement: Supplementary file 2 — Venn diagrams display the number of OTUs (A) and proteins (C) attributed to the three adaptation periods. B, D are tables showing the respective p values calculated by a pair-wise comparison to show the significant differences between the time points and diets. Figure S2. Taxonomic assessment of the samples at each of the selected experimental time points (days). The entire metaproteomic dataset (i.e. both unique and shared proteins) is considered for the fecal microbiota taxonomic assessment. Figure S3. LFQ distribution among the adaptation periods. Pie charts represent the relative distribution of the abundance index of the proteins identified in zero (A) MA (B) and EQ (C) samples. Figure S4. Functional classification of the identified proteins by their categorization into COG classes (A) and KEGG biochemical pathways (B). Only categories with a cumulative abundance higher that 1% of the total LFQ abundance index are included in the visualisation. A functional classification of the samples at all the selected experimental time points (days) is provided. Figure S5. Heat map displays a list of proteins whose abundance ratio is changing between adaptation periods of at least 5-fold. Abundance indexes of each protein in the diverse adaptation periods are shown as log LFQ. Figure S6. Protein classification into KEGG biochemical pathways. Abundance of the pathways is expressed as a relative percentage for each of the adaptation periods. The only pathways scoring at least 2.5-fold change between the adaptation periods are visualised. Figure S7. SCFA production as assessed through the metaproteomic (A and B) and conventional approach (C and D). A Abundance of the enzymes, selected as indicators of SCFA production, out of the total LFQ abundance indexes. B Distribution of the indicators for the major SCFA production, across the diverse adaptation periods. C Summary of the SCFA measurements in the zero and EQ period, on an animal basis. D Relative production of the major SC [file 40168_2017_362_MOESM2_ESM.zip › Figuree S1_Dataset of adaptation periods.pdf]

A

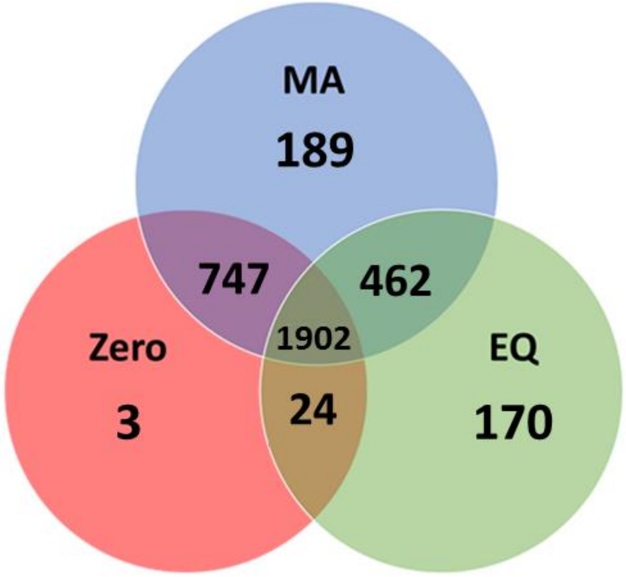

C

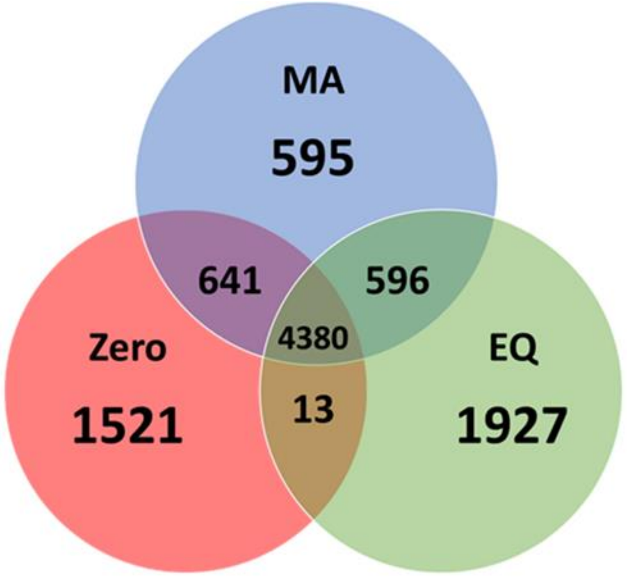

B

	period	Zero	Zero	Zero	Zero	MA	MA	MA	MA	EQ	EQ	EQ
period	diet	HD, CaP high	HD, CaP low	LD, CaP high	LD, CaP low	HD, CaP high	HD, CaP low	LD, CaP high	LD, CaP low	HD, CaP high	HD, CaP low	LD, CaP high
Zero	HD, CaP low	1.000										
Zero	LD, CaP high	0.809	1.000									
Zero	LD, CaP low	0.592	0.898	0.888								
MA	HD, CaP high	0.714	0.269	0.351	0.102							
MA	HD, CaP low	0.211	0.713	0.352	0.121	**0.026**						
MA	LD, CaP high	**0.044**	0.076	0.506	**0.041**	***0.001***	**0.003**					
MA	LD, CaP low	0.176	0.333	0.383	0.770	**0.004**	**0.012**	**0.007**				
EQ	HD, CaP high	**0.018**	**0.009**	**0.034**	**0.011**	***0.001***	***0.001***	***0.001***	***0.001***			
EQ	HD, CaP low	**0.011**	**0.014**	**0.038**	**0.015**	***0.001***	***0.001***	***0.001***	***0.001***	***0.001***		
EQ	LD, CaP high	**0.012**	**0.010**	**0.033**	**0.008**	***0.001***	***0.001***	***0.001***	***0.001***	**0.008**	**0.002**	
EQ	LD, CaP low	**0.011**	**0.019**	**0.034**	**0.010**	***0.001***	***0.001***	***0.001***	***0.001***	**0.003**	**0.002**	**0.002**

D

	period	Zero	Zero	Zero	Zero	MA	MA	MA	MA	EQ	EQ	EQ
period	diet	HD, CaP high	HD, CaP low	LD, CaP high	LD, CaP low	HD, CaP high	HD, CaP low	LD, CaP high	LD, CaP low	HD, CaP high	HD, CaP low	LD, CaP high
Zero	HD, CaP low	1.000										
Zero	LD, CaP high	0.705	0.797									
Zero	LD, CaP low	0.621	0.902	0.819								
MA	HD, CaP high	0.507	0.200	0.160	0.140							
MA	HD, CaP low	0.422	0.523	0.326	0.321	0.234						
MA	LD, CaP high	0.123	0.097	0.250	0.088	0.131	0.135					
MA	LD, CaP low	0.442	0.442	0.125	0.268	0.09	0.096	0.07				
EQ	HD, CaP high	**0.013**	**0.01**	**0.021**	**0.014**	***0.001***	***0.001***	***0.001***	***0.001***			
EQ	HD, CaP low	**0.011**	**0.017**	**0.011**	**0.014**	***0.001***	***0.001***	***0.001***	***0.001***	**0.006**		
EQ	LD, CaP high	**0.017**	**0.019**	**0.01**	**0.018**	***0.001***	***0.001***	***0.001***	***0.001***	**0.007**	**0.006**	
EQ	LD, CaP low	**0.012**	**0.015**	**0.012**	**0.013**	***0.001***	***0.001***	***0.001***	***0.001***	**0.004**	**0.037**	**0.003**
